# A New Ergosterol Analog, a New *Bis*-Anthraquinone and Anti-Obesity Activity of Anthraquinones from the Marine Sponge-Associated Fungus *Talaromyces stipitatus* KUFA 0207

**DOI:** 10.3390/md15050139

**Published:** 2017-05-16

**Authors:** Jidapa Noinart, Suradet Buttachon, Tida Dethoup, Luís Gales, José A. Pereira, Ralph Urbatzka, Sara Freitas, Michael Lee, Artur M. S. Silva, Madalena M. M. Pinto, Vítor Vasconcelos, Anake Kijjoa

**Affiliations:** 1 ICBAS-Instituto de Ciências Biomédicas Abel Salazar, Rua de Jorge Viterbo Ferreira, 228, 4050-313 Porto, Portugal; jidanoinart@gmail.com (J.N.); nokrari_209@hotmail.com (S.B.); lgales@ibmc.up.pt (L.G.); jpereira@icbas.up.pt (J.A.P.); 2 Interdisciplinary Centre of Marine and Environmental Research (CIIMAR), Terminal de Cruzeiros do Porto de Lexões, Av. General Norton de Matos s/n, 4450-208 Matosinhos, Portugal; rurbatzka@ciimar.up.pt (R.U.); freitas.srf.09@gmail.com (S.F.); madalena@ff.up.pt (M.M.M.P.); vmvascon@fc.up.pt (V.V.); 3 Department of Plant Pathology, Faculty of Agriculture, Kasetsart University, Bangkok 10240, Thailand; tdethoup@yahoo.com; 4 Instituto de Biologia Molecular e Celular (IBMC), Universidade do Porto, Rua de Jorge Viterbo Ferreira, 228, 4050-313 Porto, Portugal; 5 Department of Chemistry, University of Leicester, University Road, Leicester LE 7 RH, UK; ml34@leicester.ac.uk; 6 Departamento de Química & QOPNA, Universidade de Aveiro, 3810-193 Aveiro, Portugal; artur.silva@ua.pt; 7 Laboratório de Química Orgânica, Departamento de Ciências Químicas, Faculdade de Farmácia, Universidade do Porto, Rua de Jorge Viterbo Ferreira, 228, 4050-313 Porto, Portugal; 8 Departamento de Biologia, Faculdade de Ciências, Universidade do Porto, Rua do Campo Alegre, s/n, 4169-007 Porto, Portugal

**Keywords:** *Talaromyces stipitatus*, Trichocomaceae, anthraquinones, *bis*-anthraquinone, ergosterol derivatives, secalonic acid A, anti-obesity, zebrafish Nile red assay

## Abstract

A new ergosterol analog, talarosterone (**1**) and a new *bis*-anthraquinone derivative (**3**) were isolated, together with ten known compounds including palmitic acid, ergosta-4,6,8(14),22-tetraen-3-one, ergosterol-5,8-endoperoxide, cyathisterone (**2**), emodin (**4a**), questinol (**4b**), citreorosein (**4c**), fallacinol (**4d**), rheoemodin (**4e**) and secalonic acid A (**5**), from the ethyl acetate extract of the culture of the marine sponge-associated fungus *Talaromyces stipitatus* KUFA 0207. The structures of the new compounds were established based on extensive 1D and 2D spectral analysis, and in the case of talarosterone (**1**), the absolute configurations of its stereogenic carbons were determined by X-ray crystallographic analysis. The structure and stereochemistry of cyathisterone (**2**) was also confirmed by X-ray analysis. The anthraquinones **4a**–**e** and secalonic acid A (**5**) were tested for their anti-obesity activity using the zebrafish Nile red assay. Only citreorosein (**4c**) and questinol (**4b**) exhibited significant anti-obesity activity, while emodin (**4a**) and secalonic acid A (**5**) caused toxicity (death) for all exposed zebrafish larvae after 24 h.

## 1. Introduction

**Figure 1:**
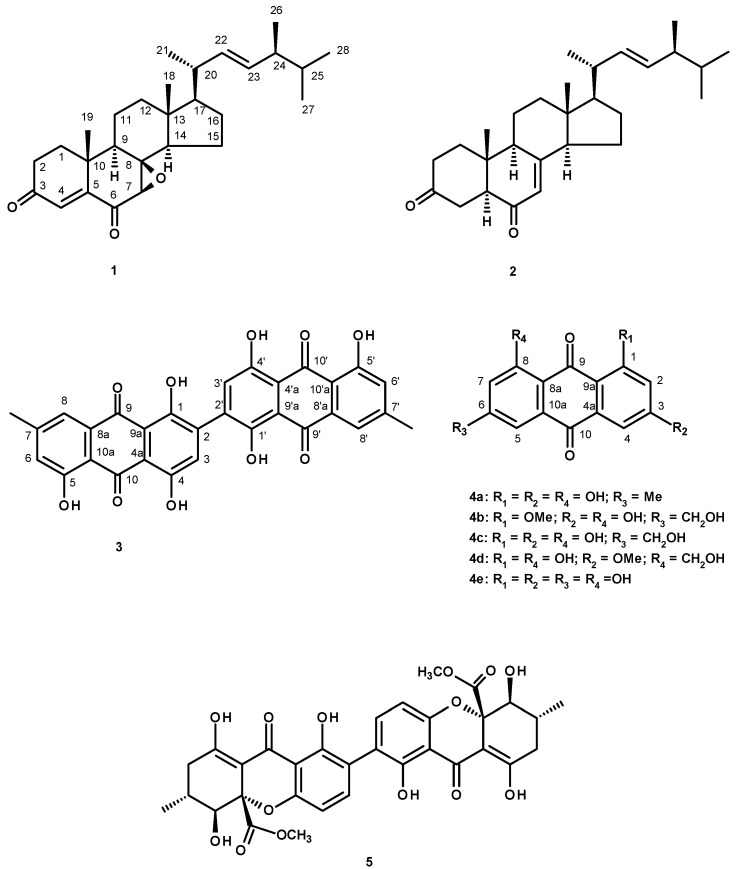
Secondary metabolites from *Talaromyces stipitatus* KUFA 0207.

The genus *Talaromyces* (Trichocomaceae), a sexual state of *Penicillium*, is important because of its ubiquity. They have been isolated from soil, plants, sponges and foods [1]. The fungi of this genus produce a wide variety of secondary metabolites including alkaloids, peptides, lactones, polyketides, and miscellaneous structural type of compounds [2]. Recently, we have reported the isolation of wortmin, *meso*-1,4-*bis*(4-methoxybenzyl)-2,3-butanediol and a new isocoumarin derivative tratenopyrone from the ethyl acetate extract of the culture of *Talaromyces tratensis* KUFA 0091, isolated from the marine sponge *Mycale* sp., collected from the Gulf of Thailand [3]. During our ongoing research on secondary metabolites from marine-derived fungi with potential anti-obesity activity, we have investigated the secondary metabolites of a Thai collection of *T. stipitatus* KUFA 0207, isolated from the marine sponge *Stylissa flabelliformis*, collected from the coral reef at Samaesarn Island in the Gulf of Thailand. The ethyl acetate extract of the culture of this fungus furnished, in addition to two new compounds including a new analog of ergosterol: talarosterone (**1**) and a new *bis*-anthraquinone derivative (**3**), palmitic acid and the previously reported ergosta-4,6,8(14),22-tetraen-3-one [4], ergosterol 5,8-endoperoxide [5], cyathisterone (**2**) [6], emodin (**4a**) [7], questinol (**4b**) [8], citreorosein (**4c**) [7], fallacinol (**4d**) [9], rheoemodin (**4e**) [10], and secalonic acid A (**5**) [11,12] (Figure 1).

Obesity is increasing at an alarming rate and only a few medications are currently on the market [13]. Recent researches demonstrated that many natural products, including secondary metabolites from plants, cyanobacteria, fungi and phytoplankton, possess anti-obesity activities [14,15]. Although many phenolic compound-containing plant extracts and several of their phenolic constituents such as flavonoids have been demonstrated to have potential as anti-obesity agents with different modes of action [15], only a few anthraquinones have been investigated for their anti-obesity potential. Huang et al. [16] have patented (US 8247001 B2) the anti-obesity product and the method of preparation of the alcohol extract of *Cassia obtusifolia* seeds, which contains the anthraquinones aurantio-obtusin, obtusifolin and their glycosides, for use in the treatment of obesity and related metabolic and liver diseases. Tzeng et al. [17], in their investigation of the anti-obesity and antihyperlipidaemic effects of emodin, have found that this anthraquinone caused dose-related reductions in the hepatic triglyceride and cholesterol contents and lowered hepatic lipid droplets accumulation in high-fat diet-fed rats. In order to investigate the potential of anthraquinone-producing marine fungi as a source of anti-obesity compounds, anthraquinones **4a**–**e** and secalonic acid A (**5**) were evaluated for their anti-obesity activity using the zebrafish Nile red assay. Zebrafish is gaining popularity as a screening system for bioactive compounds with the advantages of a small whole animal model, and higher physiological relevance over cellular in vitro models [18]. The zebrafish larvae Nile red assay is suitable to detect anti-obesogenic activities since zebrafish larvae were demonstrated to respond to known lipid regulator drugs similarly as humans [19].

## 2. Results and Discussion

**Figure 2:**
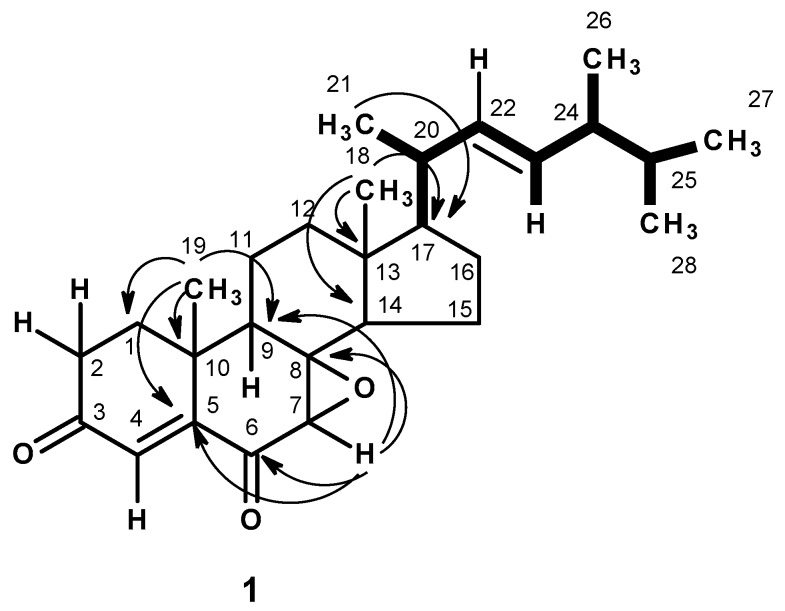
Key COSY (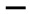) and HMBC (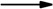) correlations of compound **1**.

The molecular formula C_28_H_40_O_3_ of compound **1**, a white crystal (mp, 145–148 °C), was established based on the (+)-HRESIMS *m*/*z* 425.3056 [M + H]^+^ (calculated 425.3056), indicating nine degrees of unsaturation. The IR spectrum showed absorption bands for conjugated ketone carbonyl (1689, 1670 cm^−1^) and olefin (1593 cm^−1^) groups. The ^1^H and ^13^C NMR spectra (Table 1, Supplementary Information, Figures S1 and S2) of **1** showed similar characteristics of ergosterol derivatives. The ^13^C NMR spectrum of **1** showed the presence of twenty-eight carbon signals which can be classified, by DEPTs and HSQC spectra (Supplementary Information, Figure S4), as two conjugated ketone carbonyl (δ_C_ 199.5 and 195.9), one quaternary sp^2^ (δ_C_ 155.0), three methine sp^2^ (δ_C_ 135.0, 132.5, 129.2), one oxyquaternary sp^3^ (δ_C_ 66.2), two quaternary sp^3^ (δ_C_ 44.3, 36.8), seven methine sp^3^ (δ_C_ 56.1, 56.0, 51.8, 43.9, 42.8, 40.0, 33.0), six methylene sp^3^ (δ_C_ 39.0, 37.1, 33.8, 27.7, 20.7, 18.7), two tertiary methyl (δ_C_ 21.8, 12.4) and four secondary methyl (δ_C_ 21.0, 20.0, 19.6, 17.6) groups. The ^1^H NMR spectrum (Table 1, Supplementary Information, Figure S1) exhibited a doublet of an olefinic proton at δ_H_ 6.66 (*J* = 0.8 Hz), a singlet of one proton at δ_H_ 3.20, two singlets of the tertiary methyls at δ_H_ 1.50 and 0.89, in addition to the proton signals of the (3*E*)-5,6-dimethylhept-3-en-2-yl side chain [δ_H_ 0.82, d, *J* = 6.8 Hz (H_3_-27), δ_H_ 0.84, d, *J* = 6.8 Hz (H_3_-28), δ_H_ 1.04, d, *J* = 6.8 Hz (H_3_-21), δ_H_ 5.15, dd, *J* = 15.3, 7.3 Hz (H-22) and δ_H_ 5.25, dd, *J* = 15.3, 8.0 Hz (H-23)]. As both H_3_-21 and the methyl singlet at δ_H_ 0.89 showed HMBC cross peaks to C-17 (δ_C_ 56.0), the methyl singlet at δ_H_ 0.89 was assigned to CH_3_-18. Therefore, another methyl singlet (δ_H_ 1.50, δ_C_ 21.8) was assigned to CH_3_-19. Since the HMBC spectrum (Table 1 and Figure 2, Supplementary Information, Figure S5) also exhibited cross peaks from both H_3_-19 and the doublet of the olefinic proton at δ_H_ 6.66 (*J* = 0.8 Hz, H-4) to the quaternary sp^2^ carbon at δ_C_ 155.0, the double bond was placed between C-4 (δ_C_ 129.2) and C-5 (δ_C_ 155.5). Moreover, H_3_-19 also showed HMBC cross peaks to the carbon signals at δ_C_ 43.9, 37.1 and 36.8, therefore, they were assigned to C-9, C-1 and C-10, respectively. On the other hand, the carbons at δ_C_ 44.3 and 51.8 were assigned to C-13 and C-14 since they both showed HMBC cross peaks to H_3_-18. Moreover, since the HMBC spectrum (Table 1 and Figure 2, Supplementary Information, Figure S5) also exhibited cross peaks from the singlet at δ_H_ 3.20 to C-5, C-14, the carbons at δ_C_ 66.2 and δ_C_ 195.9, the carbon at δ_C_ 66.2 was assigned to C-8 and one of the conjugated carbonyl (δ_C_ 195.9) was placed on C-6. This was supported by the HMBC cross peak from H-4 to C-6. Therefore, another conjugated carbonyl group (δ_C_ 199.5) must be on C-3. The fact that there was one more oxygen atom to be accounted for, along with the presence of the oxyquaternary carbon (δ_C_ 66.2), the epoxide function was placed between C-7 and C-8. Therefore, the structure of **1** was elucidated as 7,8-epoxyergosta-4,22-dien-3,6-dione.

**Figure 3:**
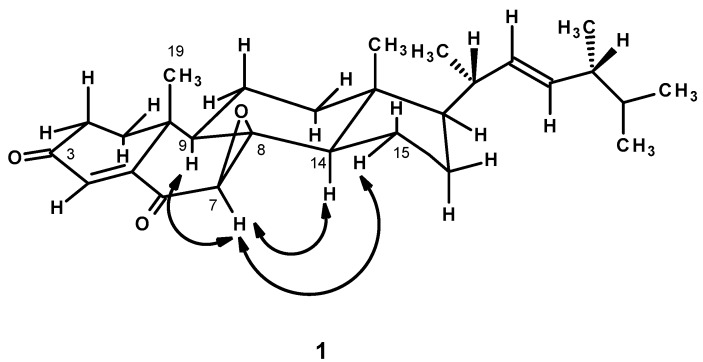
Key ROESY correlations (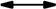) of compound **1**.

In order to determine the stereochemistry of **1**, the ROESY spectrum was obtained. As the ROESY spectrum (Table 1 and Figure 3, Supplementary Information, Figure S7) showed cross peaks from H-7 to H-9, H-14 and H-15, but not to H_3_-19, it was concluded that the epoxide ring was on the same face as CH_3_-19, i.e., the relative configuration of C-7 and C-8 is 7*R* and 8*R* respectively.

**Figure 4:**
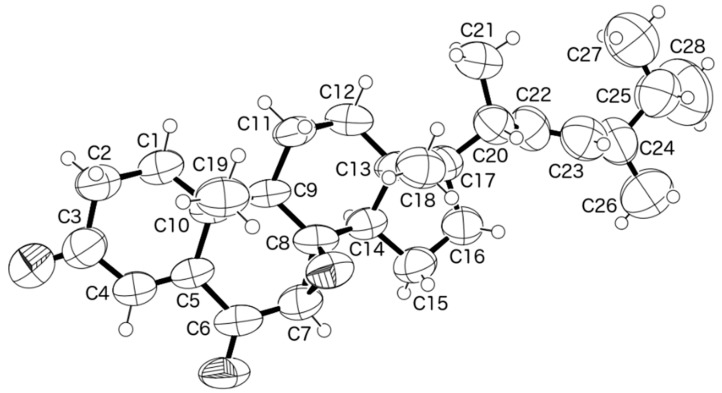
ORTEP diagram of compound **1**.

Since compound **1** was obtained in a suitable crystal, X-ray analysis was carried out, and the ORTEP view shown in Figure 4 revealed that the absolute configuration of C-7, C-8, C-9, C-10, C-13, C-14, C-17, C-20 and C-24 is 7*R*, 8*R*, 9*R*, 10*R*, 13*R*, 14*R*,17*R*, 20*S*, 24*R*. A literature search indicated that **1** has never been previously reported. Therefore, it is a new compound and was named talarosterone.

**Figure 5:**
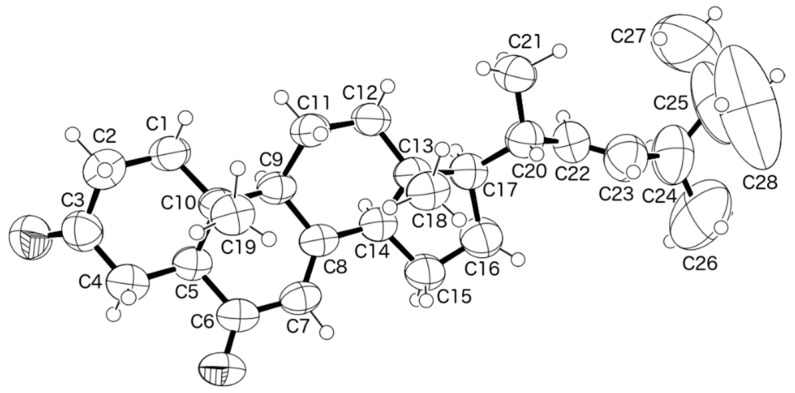
ORTEP diagram of compound **2**.

The ^1^H, ^13^C NMR, IR and HRMS data of **2** (Supplementary information, Table S1, Figures S8–S12) are compatible with those reported for cyathiserone [6]. The structure and the stereochemistry of **2** were confirmed by X-ray analysis and the ORTEP view is shown in Figure 5.

Compound **3** was isolated as a reddish orange solid (mp, 258–260 °C). The IR spectrum showed absorption bands for hydroxyl (3463 cm^−1^), conjugated ketone carbonyl (1622 cm^−1^) and aromatic (1550 cm^−1^) groups. The ^13^C NMR spectrum (Table 2, Supplementary Information, Figure S14) exhibited fifteen carbon signals which, in conjunction with DEPTS and HSQC spectra (Supplementary Information, Figure S16), can be categorized as two conjugated ketone carbonyls (δ_C_ 189.6 and 182.1), nine quaternary sp^2^ (δ_C_ 164.4, 164.3, 161.1, 148.2, 133.2, 131.3, 123.5, 113.1, 108.9), three methine sp^2^ (δ_C_ 123.6, 120.5, 107.2) and one tertiary methyl (δ_C_ 21.5) groups. The ^1^H NMR spectrum (Table 2, Supplementary Information, Figure S13) showed one singlet at δ_H_ 6.73 and two double doublets at δ_H_ 7.15 (*J* = 1.0, 0.5 Hz) and 7.28 (*J* = 1.0, 0.5 Hz) of the aromatic protons and one singlet of the tertiary methyl (δ_H_ 2.33), in addition to two singlets of the hydrogen-bonded hydroxyl groups at δ_H_ 12.04 and δ_H_ 12.79. 

**Figure 6:**
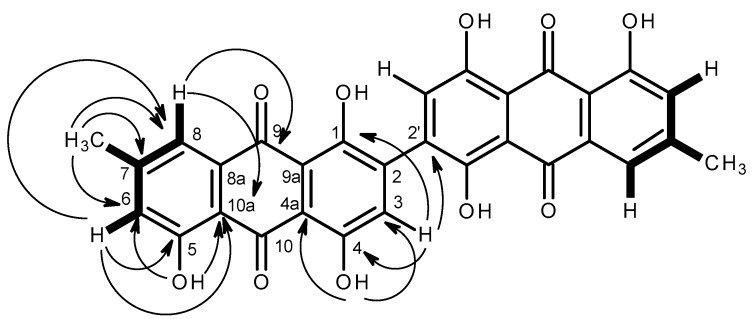
Key COSY (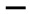) and HMBC (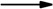) correlations of compound **3**.

The ^1^H and ^13^C NMR features suggested that **3** was a polyhydroxy methyl anthraquinone. That one of the benzene rings of **3** was a 3-hydroxy-5-methyl-1,2,3,5-tetrasubstituted was supported by the COSY correlations (Table 2 and Figure 6, Supplementary Information, Figure S15) of the aromatic protons at δ_H_ 7.15 (*J* = 1.0, 0.5 Hz, H-6) and 7.28 (*J* = 1.0, 0.5 Hz, H-8) with the methyl protons at δ_H_ 2.33 as well as by the HMBC correlations from H-6 to C-8 (δ_C_ 120.5), C-5 (δ_C_ 161.1), C-10a (δ_C_ 113.1), and CH_3_-7 (δ_C_ 21.5), from H-8 to C-6 (δ_C_ 123.6), C-10a and CH_3_-7 as well as from OH-5 (δ_H_ 12.04, s) to C-5, C-6 and C-10a (Table 2 and Figure 6, Supplementary Information, Figure S17). Since the methyl protons showed HMBC correlation with the carbon at δ_C_ 148.2, it was assigned to C-7. On the other hand, another benzene ring of **3** was 3, 6-dihydroxy-1,2,3,5,6-substituted since the HMBC spectrum (Table 2 and Figure 6, Supplementary Information, Figure S17) exhibited correlations from the aromatic proton singlet at δ_H_ 6.73 (H-3) to C-1 (δ_C_ 164.3), C-4 (δ_C_ 164.4), C-4a (δ_C_ 108.9) as well as from the hydroxyl singlet at δ_H_ 12.79 (OH-4) to C-3 (δ_C_ 107.2), C-4 (δ_C_ 164.4) and C-4a (δ_C_ 108.9). Moreover, the HMBC spectrum (Table 2 and Figure 6, Supplementary Information, Figure S17) also exhibited a cross peak from H-8 to the carbonyl carbon at δ_C_ 182.1, the latter was assigned to C-9. Since, there was no cross peak from H-3 to either of the carbonyl carbons, the carbonyl carbon at δ_C_ 189.6 was assigned to C-10. The higher chemical shift value of C-10 than that of C-9 was justified by its hydrogen bonding with both OH-4 and OH-5. By comparing the ^13^C NMR data of **3** to those of other anthraquinones isolated from this fungus, i.e., emodin (**4a**), citreorosein (**4c**), fallacinol (**4d**) and rheoemodin (**4e**), the carbons at δ_C_ 131.3 and δ_C_ 133.2 were assigned to C-8a and C-9a, respectively. Therefore, the structure of **3** was tentatively established as 7-methyl-1,4,5-trihydroxy-9,10-anthraquinone. However, this structure (C_15_H_9_O_5_) was not complete since there was no substituent on C-2 (δ_C_ 123.5) to be accounted for. Since the (+)-HRESIMS of **3** gave the [M + H]^+^ peak at *m*/*z* 539.0942 [M + H]^+^, corresponding to C_30_H_19_O_10_ (calculated 539.0978), the molecular formula of **3** was C_30_H_18_O_10_ (twenty-two degrees of unsaturation). Therefore, the structure of **3** was a dimer of 7-methyl-1,4,5-trihydroxy-9,10-anthraquinone. This structure was also supported by the HMBC correlation from H-2 to the quaternary sp^2^ carbon at δ_C_ 123.5 (C-2′). Therefore, the structure of **3** was established as 2, 2′-*bis*-(7-methyl-1,4,5-trihydroxy-anthracene-9,10-dione).

Literature search revealed that Tan et al. [20] have reported isolation of the structurally similar *bis*-anthraquinone, named 2240A, from an unidentified endophytic fungus (strain no 2240), isolated from the estuarine mangrove from the South China Sea Coast. The structure of 2240A is also a dimer of 7-methyl-1,4,5-trihydroxy-9,10-anthraquinone; however, the two anthraquinone monomers are linked between C-3 and C-3′ instead of C-2 and C-2′ as in **3**. Surprisingly, the ^1^H and ^13^C NMR chemical shift values reported for 2240A by Tan et al. [20] were very similar to those of **3**. Although the structures of **3** and 2240A cannot be distinguished by HMBC correlations, careful analysis of the ^1^H and ^13^C NMR data of compound 2240A revealed that Tan et al. [20] assigned the chemical shift values to some of the carbons differently from what we assigned for **3**. Tan et al. [20] assigned the carbon signal at δ_C_ 108.7 to C-9a/9a′ (i.e., C-14/14′ for the numbering used by Tan et al.) instead of C-4a/4′a (i.e., C-13/13′ for the numbering used by Tan et al.) and the carbon signal at δ_C_ 131.8 to C-4a/4′a (i.e., C-13/C-13′ for the numbering used by Tan et al.) instead of C-9a/9a′ (i.e., C-13/C-13′ for the numbering used by Tan et al.). This assignment seems to be incorrect since the chemical shift values of C-4a and C-10a of a series of anthraquinones with the hydroxyl groups on C-4 and C-5 isolated from this extract, i.e., emodin (**4a**), citreorosein (**4c**), fallacinol (**4d**) and rheoemodin (**4e**), are ca. 108 and 115 ppm, which is far from 133 ppm proposed by Tan et al. [19]. Consequently, we are convinced that the structure proposed for compound 2240A is not correct. Interestingly, Tan et al. [19] reported 2240A as dextrorotatory, displaying [α${]}_{\text{D}}^{20}$ +62.50 (*c* 0.08, dioxin). On the contrary, **3** is levorotatory having [α${]}_{\text{D}}^{20}$ −40 (*c* 0.05, dioxin) and −100 (*c* 0.05, MeOH), respectively. Since **3** can be considered as a bridged biphenyl, it can have a phenomenon of atropisomerism due to a restricted rotation of the phenyl rings around the C-2/C-2′ bond. Therefore, **3** and compound 2240A, previously reported by Tan et al. [19] are different and could probably be atropisomers.

The structure of **3** can be viewed as a dimer of helminthosporin, an anthraquinone previously isolated from the subterranean stems of *Aloe saponaria* Haw. [21], and also from the roots of *Berchemia floribunda* [22].

The structures of the other known compounds, i.e., palmitic acid, ergosta-4,6,8(14),22-tetraen-3-one, ergosterol-5,8-endoperoxide, emodin (**4a**), questinol (**4b**), citreorosein (**4c**), fallacinol (**4d**), rheoemodin (**4e**) and secalonic acid A (**5**) were elucidated by analysis of their ^1^H, ^13^C NMR and HRMS spectra, and a rotation when the compounds have stereogenic carbons as well as by comparison of these spectroscopic data with those reported in the literature.

**Figure 7:**
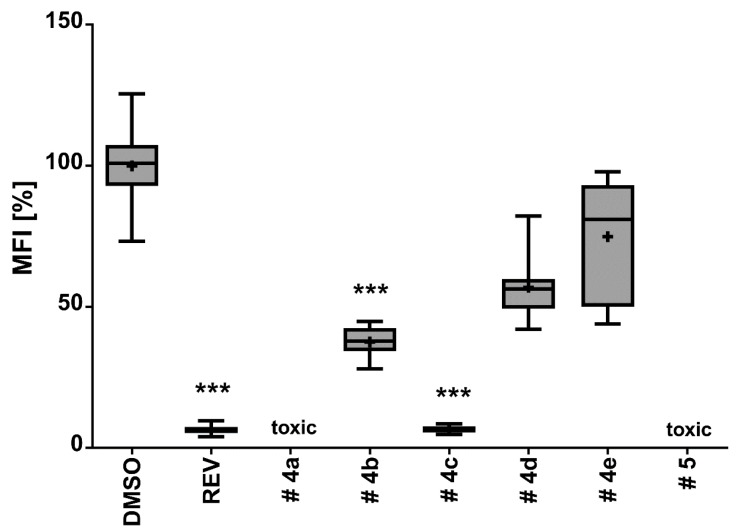
Anti-obesity activity of compounds **4a**–**e** and **5** in the zebrafish larvae Nile red assay. The solvent control had 0.1% DMSO and the positive control received 50 µM resveratrol (REV). Values are presented as mean fluorescence intensity (MFI) relative to the DMSO group, and are derived from 10 to 12 individual larvae per treatment group. Statistical differences to the solvent control are indicated with asterisks, *** = *p* < 0.001.

**Figure 8:**
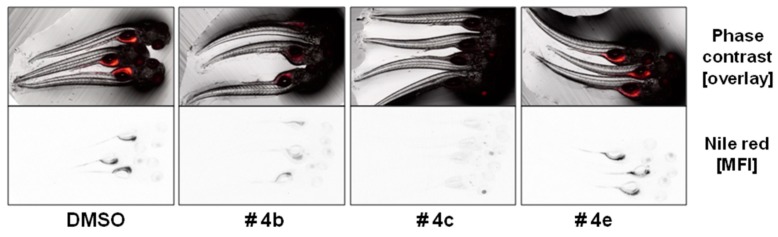
Representative images of the zebrafish Nile red assay. The upper images show the overlay of the fluorescence and phase contrast; the lower images show the mean fluorescence intensity (MFI) given as black and white picture. DMSO, solvent control 0.1%.

Emodin (**4a**), questinol (**4b**), citreorosein (**4c**), fallacinol (**4d**), rheoemodin (**4e**), and secalonic acid A (**5**) were tested for their anti-obesity activity using the zebrafish Nile red assay. The results showed that only the anthraquinones questinol (**4b**) and citreorosein (**4c**) had significant anti-obesity activity. Questinol (**4b**) and citreorosein (**4c**) reduced >60% and >90% of the stained lipids with the IC_50_ values of 0.95 and 0.17 µM, respectively. The positive control resveratrol (REV) had an IC_50_ value of 0.6 µM (Figure 7). Interestingly, emodin (**4a**) and secalonic acid A (**5**) caused toxicity (death) for all exposed zebrafish larvae after 24 h, while fallacinol (**4d**) and rheoemodin (**4e**) did not have any significant effects (Figure 8).

It is interesting to observe that questinol (**4b**), citreorosein (**4c**) and fallacinol (**4d**) are structurally similar, all having a hydroxymethyl group on C-6 and a hydroxyl group on C-8. Replacing the hydroxyl group on C-1 by a methoxyl group, as in questinol (**4b**), diminishes the activity whereas replacing the hydroxyl group on C-3 with a methoxyl group, as in fallacinol (**4d**), completely removes the anti-obesity activity. Therefore, it seems that the hydroxymethyl group on C-6 and the hydroxyl groups on C-3 and C-8 are necessary for the anti-obesity activity of the polyhydroxy anthraquinones.

## 3. Experimental Section

### 3.1. General Experimental Procedures

Melting points were determined on a Bock monoscope and are uncorrected. Optical rotations were measured on an ADP410 Polarimeter (Bellingham + Stanley Ltd., Tunbridge Wells, Kent, UK). Infrared spectra were recorded in a KBr microplate in a FTIR spectrometer Nicolet iS10 from Thermo Scientific (Waltham, MA, USA) with Smart OMNI-Transmission accessory (Software 188 OMNIC. ^1^H and ^13^C NMR spectra were recorded at ambient temperature on a Bruker AMC instrument (Bruker Biosciences Corporation, Billerica, MA, USA) operating at 300.13 and 75.4 Hz or at 500.13 and 125.4 MHz, respectively. High resolution mass spectra were measured with a Waters Xevo QToF mass spectrometer (Waters Corporations, Milford, MA, USA) coupled to a Waters Aquity UPLC system. A Merck (Darmstadt, Germany) silica gel GF_254_ was used for preparative TLC, and a Merck Si gel 60 (0.2–0.5 mm) was used for column chromatography.

### 3.2. Fungal Material

The strain KUFA 0207 was isolated from the marine sponge *Stylissa flabelliformis*, which was collected by scuba diving at a depth of 10–15 m, from the coral reef at Samaesarn Island (12°34′36.64″ N 100°56′59.69″ E) in the Gulf of Thailand, Chonburi Province, in April 2014. The sponge was washed with 1% sodium hypochlorite solution for 1 min, followed by sterilized seawater three times, and then dried on sterile filter paper under a laminar flow hood, cut into small pieces (5 × 5 mm), and placed on malt extract agar (MEA) plates containing 70% seawater and 300 mg/L of streptomycin sulfate. The plates were incubated at 28 °C for seven days, after which the hyphal tips were transferred onto a slant MEA and maintained as pure culture for further identification. The fungus was identified as *Talaromyces stipitatus* C.R. Benj., based on morphological characteristics, and was also confirmed by analysis sequence of the internal transcribed spacer (ITS) gene according to the procedure previously described by us [23]. Its gene sequences were deposited in GenBank with accession number KU500028. The pure cultures were deposited as KUFA 0207 at Kasetsart University Fungal Collection, Department of Plant Pathology, Faculty of Agriculture, Kasetsart University, Bangkok, Thailand. The fungus was cultured for one week at 28 °C in 10 Petri dishes (i.d. 90 mm) containing 25 mL of MEA. In order to obtain the mycelial suspension, the mycelial plugs were transferred to two 500 mL Erlenmeyer flasks containing 200 mL of potato dextrose broth, and then incubated on a rotary shaker at 120 rpm at 28 °C for one week. Fifty 1000 mL Erlenmeyer flasks, each containing 300 g of cooked rice, were autoclaved at 121 °C for 15 min, and then inoculated with 20 mL of mycelial suspension of *T. stipitatus* and incubated at 28 °C for 30 days, after which the moldy rice was macerated in ethyl acetate (25 L total) for seven days, and then filtered. The ethyl acetate solution was concentrated under reduced pressure to yield 65.6 g of crude ethyl acetate extract.

### 3.3. Extraction and Isolation

The crude ethyl acetate (35 g) was applied on a column of silica gel (335 g), and eluted with mixtures of petrol-CHCl_3_ and CHCl_3_-Me_2_CO, 250 mL fractions were collected as follows: Frs 1–133 (petrol-CHCl_3_, 1:1), 134–249 (petrol-CHCl_3_, 3:7), 250–314 (petrol-CHCl_3_, 1:9), 315–440 (CHCl_3_-Me_2_CO, 9:1), 441–511 (CHCl_3_-Me_2_CO, 7:1), 512–546 (CHCl_3_-Me_2_CO, 1:1). Frs 156–170 were combined (382.7 mg) and crystallized in methanol to give palmitic acid (20 mg). Frs 171–182 were combined (370 mg) and purified by TLC (silica gel G_254_, CHCl_3_-petrol-EtOAc-HCO_2_H, 16:3:1:0.01) to give **1** (10.5 mg), palmitic acid (21.7 mg) and ergosta-4,6,8(14),22-tetraen-3-one (23.3 mg). Frs 220–250 were combined (201.5 mg) and crystallized in MeOH to give ergosta-4,6,8(14),22-tetraen-3-one (19.6 mg). Frs 315–317 were combined (4.53 g) and applied over a column chromatography of silica gel (35 g) and eluted with mixture of petrol-CHCl_3_ and CHCl_3_-Me_2_CO, wherein 250 mL fractions were collected as follows: sfrs 1–101 (petrol-CHCl_3_, 1:1), 102-170 (petrol-CHCl_3_, 3:7), 171–296 (petrol-CHCl_3_, 1:9), 297–311 (CHCl_3_-Me_2_CO, 9:1). Sfrs 19–38 were combined (329 mg) and applied over a column chromatography of Sephadex LH-20 (10 g) and eluted with a 1:1 mixture of CHCl_3_:MeOH, to give forty 15 mL ssfrs. Ssfrs 8–37 were combined and purified by TLC (silica gel G_254_, CHCl_3_-petrol-EtOAc-HCO_2_H, 80:16.5:3.5:0.1) to give **2** (12.2 mg). Sfrs 229–296 were combined (179 mg) and purified by TLC (silica gel G_254_, CHCl_3_-petrol-EtOAc-HCO_2_H, 80:15:5:0.1) to give emodin (**4a**, 10 mg). Frs 318–330 were combined (3.77 g) and applied over a column chromatography of silica gel (100 g) and eluted with a mixture of petrol-CHCl_3_ and CHCl_3_-Me_2_CO, wherein 100 mL fractions were collected as follows: sfrs 1–118 (petrol-CHCl_3_, 1:1), 199–277 (petrol-CHCl_3_, 3:7), 278–289 (petrol-CHCl_3_, 1:9), 290–471 (CDCl_3_), 472–589 (CHCl_3_-Me_2_CO, 9:1), 590–633 (CHCl_3_-Me_2_CO, 7:3). Sfrs 238–395 were combined (473.7 mg) and crystallized in a mixture of CHCl_3_ and petrol to give ergosterol-5,8-endoperoxide (22 mg). Sfrs 477 (146.2 mg) was crystallized in a mixture of CHCl_3_ and petrol to give a reddish orange solid of **3** (9 mg). Sfrs 510–524 were combined (53 mg) and crystallized in CHCl_3_ to give yellow solid of fallacinol (**4d**, 21 mg). Frs 332–345 were combined (60.0 mg) and crystalized in a mixture of CHCl_3_ and MeOH to give 15.2 mg of secalonic acid A. Frs 355–365 were combined (103.0 mg) and crystalized in a mixture of CHCl_3_ and MeOH to an additional 10.5 mg of secalonic acid A, whereas the mother liquor was further purified by TLC (silica gel G_254_, CHCl_3_-Me_2_CO-HCO_2_H, 4:1:0.01) to give 8.5 mg of citreorosein (**4c**). Frs 392–440 were combined (316 mg) and crystallized in Me_2_CO to give questinol (**4b**, 10.7 mg). Frs 443–450 were combined and crystallized in Me_2_CO to give rheoemodin (**4e**, 2.5 mg).

#### 3.3.1. Talarosterone (**1**): (7*R*,8*R*)-Epoxyergosta-4,22-dien-3,6-dione

White crystal; mp 146–148 °C; [α${]}_{\text{D}}^{20}$ +204 (*c* 0.04, CHCl_3_); IR (KBr) υ_max_ 2956, 2873, 1689, 1670, 1593, 1458, 1411, 1276, 1260 cm^−1^; For ^1^H and ^13^C spectroscopic data (CDCl_3_, 300.13 and 75.14 MHz), see Table 1; (+)-HRESIMS *m*/*z* 425.3056 [M + H]^+^ (calcd. for C_28_H_41_O_3_, 425.3056).

#### 3.3.2. *Bis*(1,4,5-Trihydroxy-7-methylanthraquinone) (**3**)

Reddish orange solid; mp 258–260 °C; [α${]}_{\text{D}}^{20}$ −100 (*c* 0.05, MeOH) and −40 (*c* 0.05, dioxin); IR (KBr) υ_max_ 3463, 2359, 1622, 1550, 1480, 1457, 1247, 1207 cm^−1^; For ^1^H and ^13^C spectroscopic data (CDCl_3_, 500.13 and 75.4 MHz), see Table 2; (+)-HRESIMS *m*/*z* 539.0942 [M + H]^+^ (calcd. for C_30_H_19_O_10_, 539.0978).

### 3.4. X-ray Crystal Structure of Compounds ***1*** and ***20***

Diffraction data were collected at 293 K with a Gemini PX Ultra equipped with CuK_α_ radiation (Oxford Diffraction, Abingdon, Oxfordshire, UK) (λ = 1.54184 Å). The structures were solved by direct methods using SHELXS-97 and refined with SHELXL-97 [24]. Carbon and oxygen atoms were refined anisotropically. Hydrogen atoms were either placed at their idealized positions using appropriate HFIX instructions in SHELXL, and included in subsequent refinement cycles, or were directly found from difference Fourier maps and were refined freely with isotropic displacement parameters. Full details of the data collection and refinement and tables of atomic coordinates, bond lengths and angles, and torsion angles have been deposited with the Cambridge Crystallographic Data Centre (CCDC).

Talarosterone (**1**). Crystals were orthorhombic, space group C222_1_, cell volume 5091.3(10) Å^3^ and unit cell dimensions *a* = 8.4602(5) Å, *b* = 12.407(2) Å and *c* = 48.504(5) Å. The refinement converged to *R* (all data) = 9.72% and *wR*_2_ (all data) = 15.83%. CCDC 1527335.

Cyathiserone (**2**). Crystals were monoclinic, space group P2_1_, cell volume 1278.36(8) Å^3^ and unit cell dimensions *a* = 6.6142(2) Å, *b* = 6.46731(19) Å and *c* = 29.8941(13) Å and angle β = 91.419(4)° (uncertainties in parentheses). The refinement converged to *R* (all data) = 6.96% and *wR*_2_ (all data) = 14.86%. CCDC 1527349.

### 3.5. Anti-Obesity Assay

Compounds **4a**–**e** and **5** were dissolved in DMSO at a concentration of 5 mM and stored at −20 °C until analyses. Anti-obesity activity of the compounds was analyzed with the zebrafish Nile red assay as described in Jones et al. [18] with some modifications. Zebrafish adults and larvae were maintained under standard conditions at 28 °C as defined in the zebrafish book available at the ZFIN database (https://zfin.org/). In brief, zebrafish embryos were raised from 1 DPF (days post fertilization) in egg water (60 µg/mL marine sea salt dissolved in distilled H_2_O) with 200 µM PTU (1-phenyl-2-thiourea) to inhibit pigmentation. From 3 DPF to 5 DPF, zebrafish larvae were exposed to compounds at a final concentration of 5 µM with daily renewal of water and compounds in a 24-well plate with a density of 10–12 larvae/well. A solvent control (0.1% DMSO) and positive control (REV, resveratrol, final concentration 50 µM) were included in the assay. Lipids were stained with Nile red overnight at the final concentration of 10 ng/mL. For imaging, the larvae were anaesthetized with tricaine (MS-222, 0.03%) for 5 min and fluorescence analyzed with a fluorescence microscope (Leica DM6000B, Wetzlar, Germany). Fluorescence intensity was quantified in individual zebrafish larvae by ImageJ (http://rsb.info.nih.gov/ij/index.html).

## 4. Conclusions

The ethyl acetate extract from the culture of the marine sponge-associated fungus *Talaromyces stipitatus* KUFA 0207 furnished a new ergosterol derivative (**1**), a new *bis*-anthraquinone derivative (**3**), and ten known compounds including palmitic acid, ergosta-4,6,8(14),22-tetraen-3-one, ergosterol-5,8-endoperoxide, cyathisterone (**2**), and the previously reported anthraquinones emodin (**4a**), questinol (**4b**), citreorosein (**4c**), fallacinol (**4d**), rheoemodin (**4e**), as well as secalonic acid A (**5**). The stereochemistry of the stereogenic carbons of compounds **1** and **2** was established by X-ray analysis. Compounds **4a**–**e** and **5** were tested for their anti-obesity activity by the zebrafish Nile red assay using resveratrol as a positive control. Citreorosein (**4c**) exhibited strong anti-obesity activity (IC_50_ = 0.17 μM), while questinol (**4b**) showed moderate activity (IC_50_ = 0.95 μM) in this test system. Analysis of the structures of the anthraquinones tested revealed that the hydroxymethyl group on C-6 and the hydroxyl groups on C-3 and C-8 are required for the anti-obesity activity. The results obtained from this study suggested that marine-derived fungi of the genus *Talaromyces* can be a promising source of compounds with potential anti-obesity activity.

## Figures and Tables

**Table 1:** ^1^H and ^13^C NMR (CDCl_3_, 300.13 MHz and 75.4 MHz) and HMBC assignment for **1**.

Position	δ_C_, Type	δ_H_, (*J* in Hz)	COSY	ROESY	HMBC
1α	37.1, CH_2_	1.81, m			
β		2.18, m			
2α	33.8, CH_2_	2.50,m	H-1		
β		2.64, dd (17.9, 7.2)	H-1		
3	199.5, CO	-			
4	129.2, CH	6.66, d (0.8)	-		C-2, 3, 5
5	155.0, C	-			
6	195.9, CO	-			
7	56.1, CH	3.20, s	-	H-9, 14, 15	C-5, 6, 8, 14
8	66.2, C	-			
9	43.9, CH	1.90, m			
10	36.8, C	-			
11	20.7, CH_2_	1.85, m			
12	39.0, CH_2_	1.21, m; 1.40, m			
13	44.3, C	-			
14	51.8, CH	1.94, m			
15	18.7, CH_2_	1.25, m			
16	27.7, CH_2_	1.34, m; 1.75, m			
17	56.0, CH	1.30, m			
18	12.4, CH_3_	0.89, s			C-12, 13, 14, 17
19	21.8, CH_3_	1.50, s	-	1β, 2β	C-1, 5, 9
20	40.0, CH	2.04, m			
21	21.0 CH_3_	1.04, d (6.8)	H-20		C-17, 20, 22
22	135.0, CH	5.15, dd (15.3, 7.3)	H-20, 23		C-21, 24
23	132.5, CH	5.25, dd (15.3, 8.0)	H-22, 24		C-20, 24, 25, 26
24	42.8, CH	1.85, m			C-22, 23
25	33.0, CH	1.48, m			
26	17.6, CH_3_	0.92, d (6.8)	H-24		C-23, 24, 25
27	20.0, CH_3_	0.82, d (6.8)	H-25		C-24, 25, 28
28	19.6 CH_3_	0.84, d (6.8)	H-25		C-24, 25, 27

**Table 2:** ^1^H and ^13^C NMR (DMSO, 500.13 MHz and 125.4 MHz) and HMBC assignment for **3**.

Position	δ_C_, Type	δ_H_, (*J* in Hz)	COSY	HMBC
1 (1′)	164.3, C	-		
2 (2′)	123.5, C	-		
3 (3′)	107.2, CH	6.73, s	-	C-1 (1′), 4 (4′), 2 (2′), 4a (4′a)
4 (4′)	108.9, C	-		
4a (4′a)	108.9, C	-		
5 (5′)	161.1, C	-		
6 (6′)	123.6, CH	7.15, dd (1.0, 0.5)	H-8 (8′), Me-7 (7′)	C-5 (5′), 7 (7′), 8 (8′), 10a (10′a)
7 (7′)	148.2, C	-		
8 (8′)	120.5, CH	7.28, dd (1.0, 0.5)	H-6 (6′), Me-7 (7′)	C-6 (6′), 7 (7′), 9 (9′), 10a (10′a)
8a (8′a)	131.3, C *	-		
9 (9′)	182.1, CO	-		
9a (9′a)	133.2, C *	-		
10 (10′)	189.6, CO	-		
10a (10′a)	113.1, C	-		
CH_3_-7 (7′)	21.5, CH_3_	2.33, s	H-6 (6′), 8 (8′)	C-6 (6′), 7 (7′), 8 (8′)
OH-4 (4′)	-	12.79, s	-	C-3 (3′), 4 (4′), 4a (4′a)
OH-5 (5′)	-	12.04, s	-	C-5 (5′), 6 (6′), 10a (10′a)

* can be interchanged.

## Supplementary Materials

The following are available online at www.mdpi.com/1660-3397/15/5/139/s1.
