# Supplementary material for: A New Ergosterol Analog, a New Bis-Anthraquinone and Anti-Obesity Activity of Anthraquinones from the Marine Sponge-Associated Fungus Talaromyces stipitatus KUFA 0207
Source: Mar Drugs. 2017 May 16;15(5):139. doi: 10.3390/md15050139 (PMC5450545; doi:10.3390/md15050139)
Supplement: Supplementary file 1 [file marinedrugs-15-00139-s001.pdf]

Supplementary Information

A New Ergosterol Analogue, a New Bis-Anthraquinone and Anti-obesity Activity of Anthraquinones from the Marine Sponge-Associated Fungus *Talaromyces stipitatus* KUFA 0207

Jidapa Noinart [1], Suradet Buttachon [1, 2], Tida Dethoup [3], Luís Gales [1, 4], José A. Pereira [1, 2], Ralph Urbatzka [2], Sara Freitas [2], Michael Lee [5], Artur M.S. Silva [6], Madalena M. M. Pinto [2, 7], Vítor Vasconcelos [2, 8], Anake Kijjoa [1, 2]*

[1] *ICBAS*-Instituto de Ciências Biomédicas Abel Salazar, Rua de Jorge Viterbo Ferreira, 228, 4050-313 Porto, Portugal. E-mail: jidanoinart@gmail.com (JN), jpereira@icbas.up.pt (JAP).

[2] Interdisciplinary Centre of Marine and Environmental Research (CIIMAR), Terminal de Cruzeiros do Porto de Lexões, Av. General Norton de Matos s/n, 4450-208, Matosinhos, Portugal. E-mail: nokrari_209@hotmail.com (SB), ralph.urbatzka.ciimar@gmail.com (RU), freitas.srf.09@gmail.com (SF), vmvascon@fc.up.pt (VV).

[3] Department of Plant Pathology, Faculty of Agriculture, Kasetsart University, Bangkok 10240, Thailand. E-mail: tdethoup@yahoo.com.

[4] Instituto de Biologia Molecular e Celular (IBMC), Universidade do Porto, Rua de Jorge Viterbo Ferreira, 228, 4050-313 Porto, Portugal. E-mail: lgales@ibmc.up.pt.

[5] Department of Chemistry, University of Leicester, University Road, Leicester LE 7 RH, UK. E-mail: ml34@leicester.ac.uk.

[6] Departamento de Química & QOPNA, Universidade de Aveiro, 3810-193 Aveiro, Portugal. E-mail: artur.silva@ua.pt.

[7] Laboratório de Química Orgânica, Departamento de Ciências Químicas, Faculdade de Farmácia, Universidade do Porto, Rua de Jorge Viterbo Ferreira, 228, 4050-313 Porto, Portugal. E-mail: madalena@ff.up.pt.

[8] Departamento de Biologia, Faculdade de Ciências, Universidade do Porto, Rua do Campo Alegre, s/n, 4169-007 Porto, Portugal

* To whom correspondence should be addressed, E-mail: ankijjoa@icbas.up.pt; Tel.:+351-220428331. Fax: +351-22-2062232.

Figure S1. ^{1}H NMR spectrum of compound **1** ($CDCl_3$, 300.13 MHz).

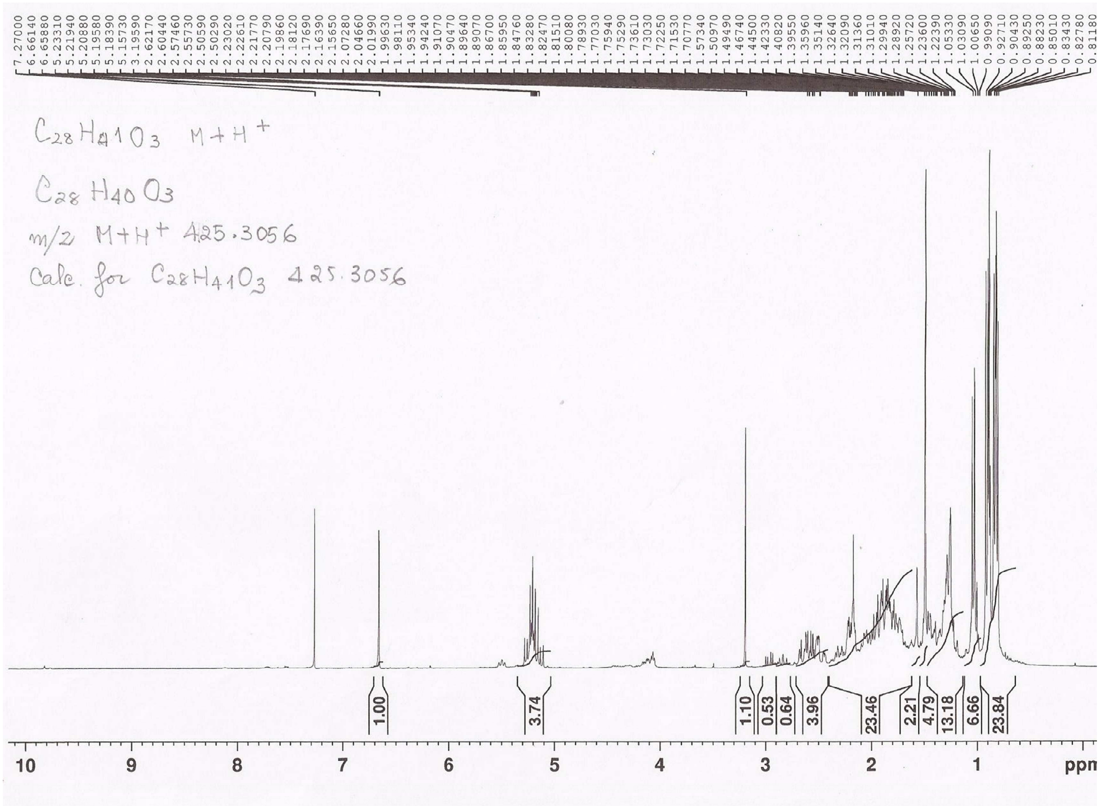

Figure S2. ^{13}C NMR spectrum of compound **1** ($CDCl_3$, 75.4 MHz).

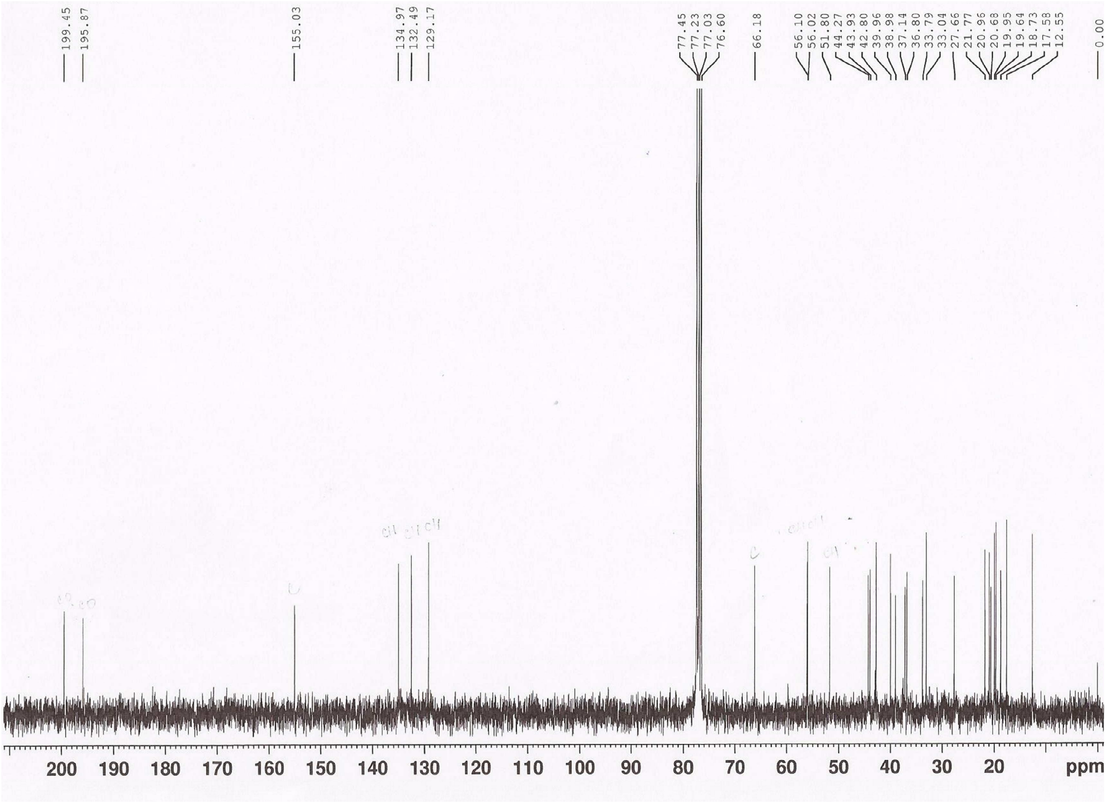

Figure S3. COSY spectrum of compound **1** ($CDCl_3$, 300.13 MHz).

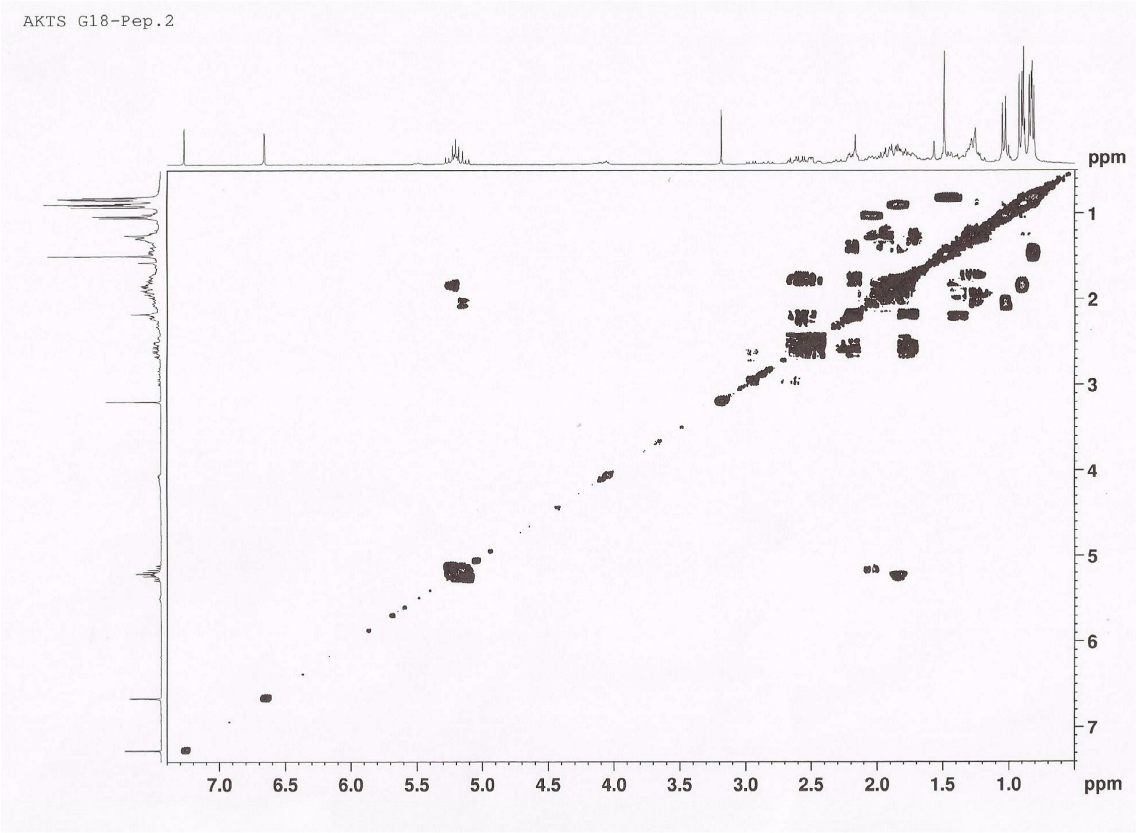

Figure S4. HSQC spectrum of compound **1** ($CDCl_3$, 300.13 MHz).

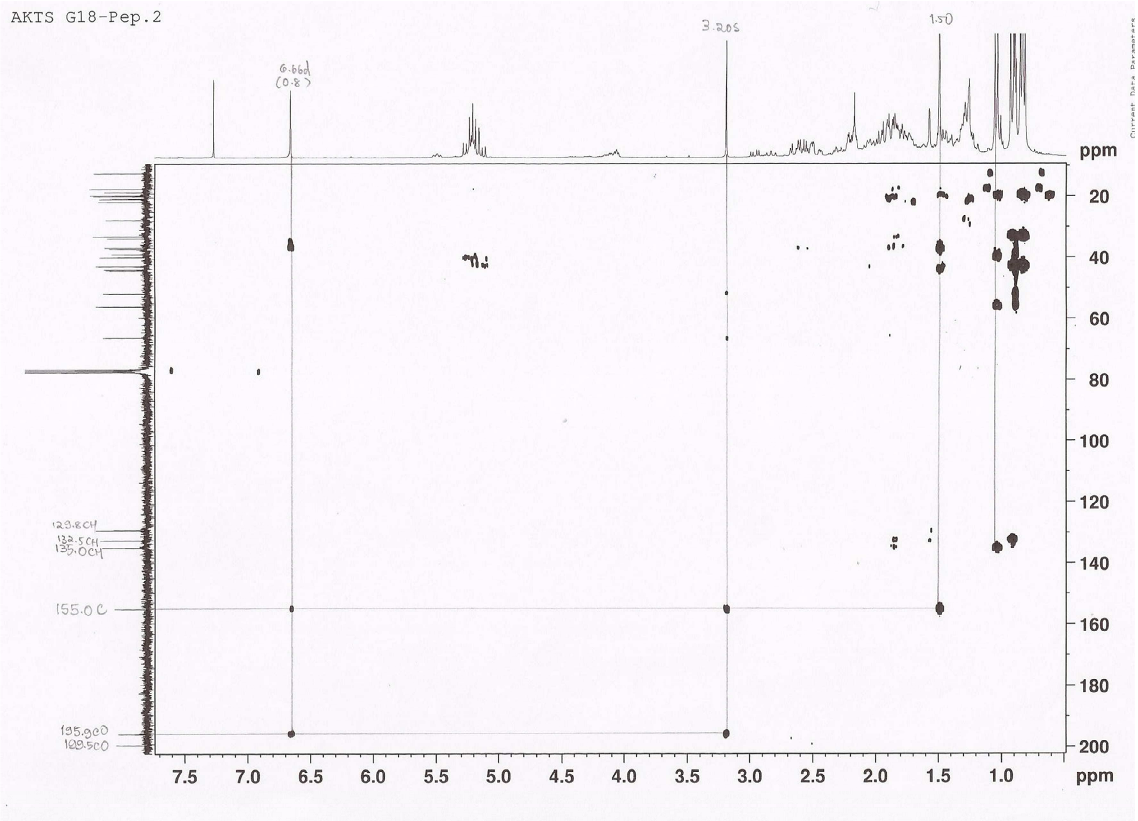

Figure S5. HMBC spectrum of compound **1** ($CDCl_3$, 300.13 MHz).

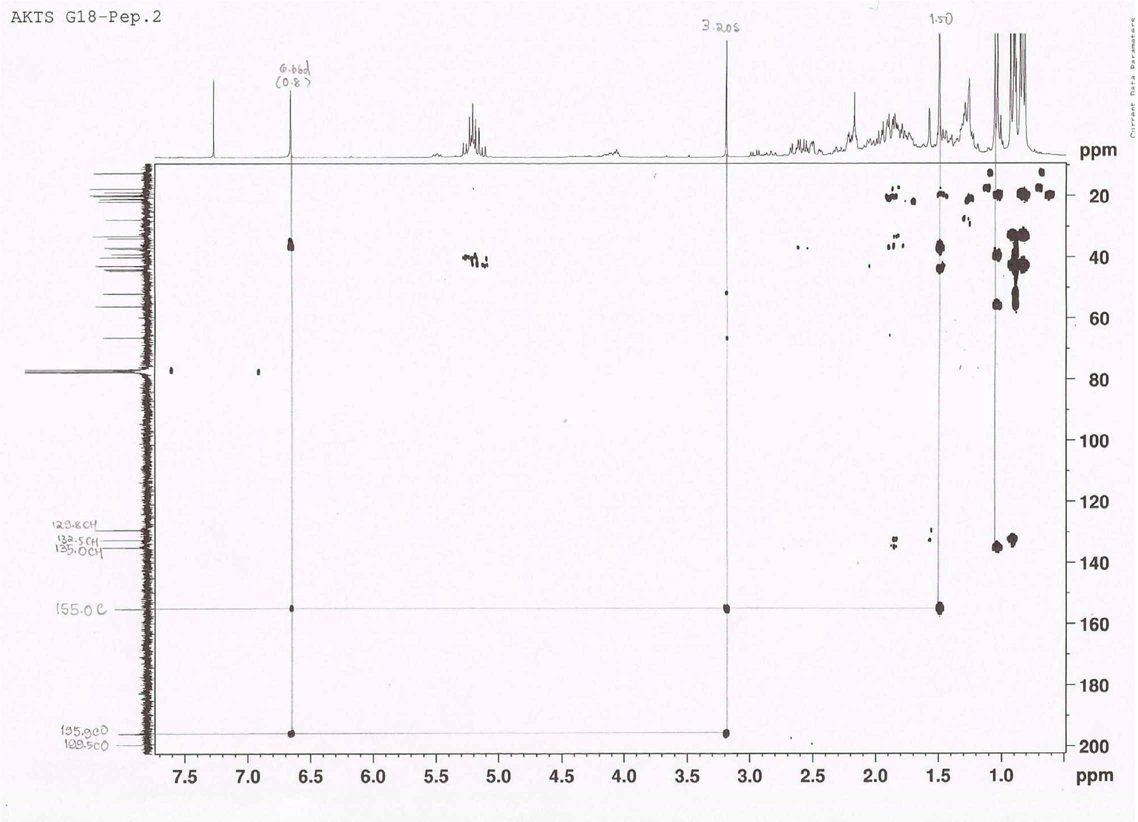

Figure S6. NOESY spectrum of compound **1** ($CDCl_3$, 500.13 MHz).

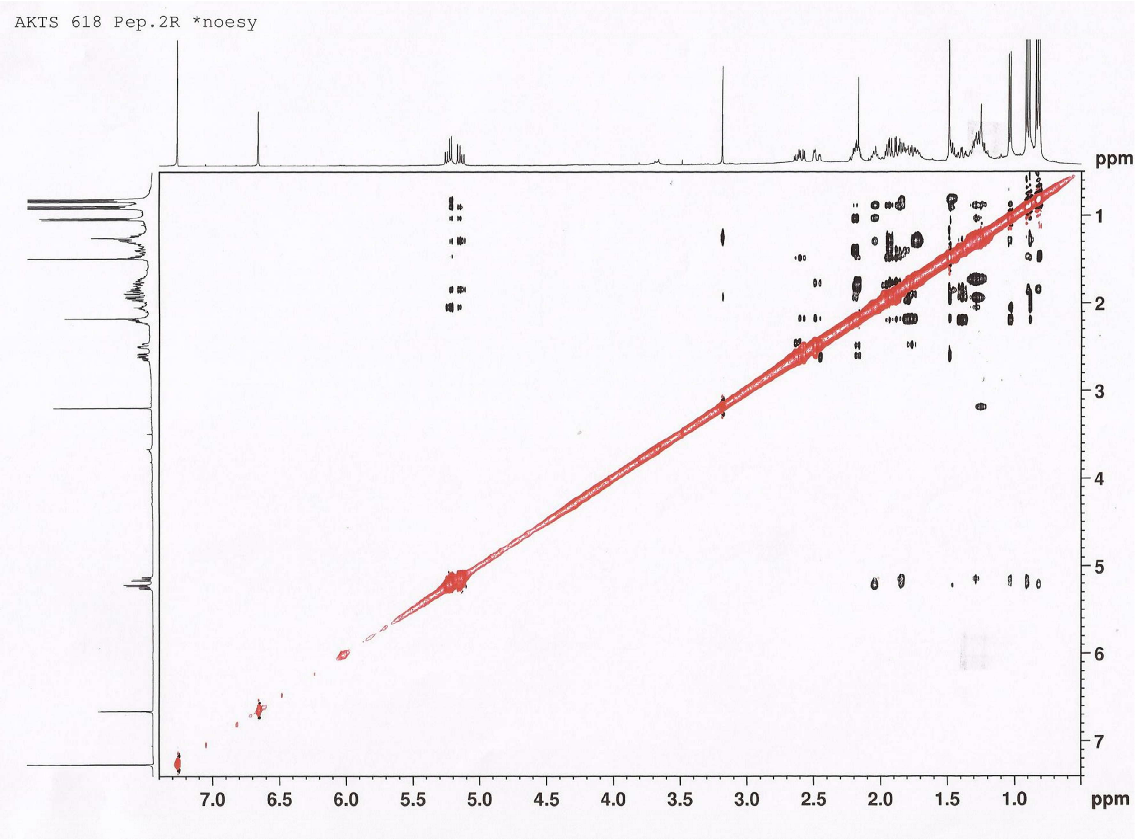

Figure S7. ROESY spectrum of compound **1** ($CDCl_3$, 500.13 MHz).

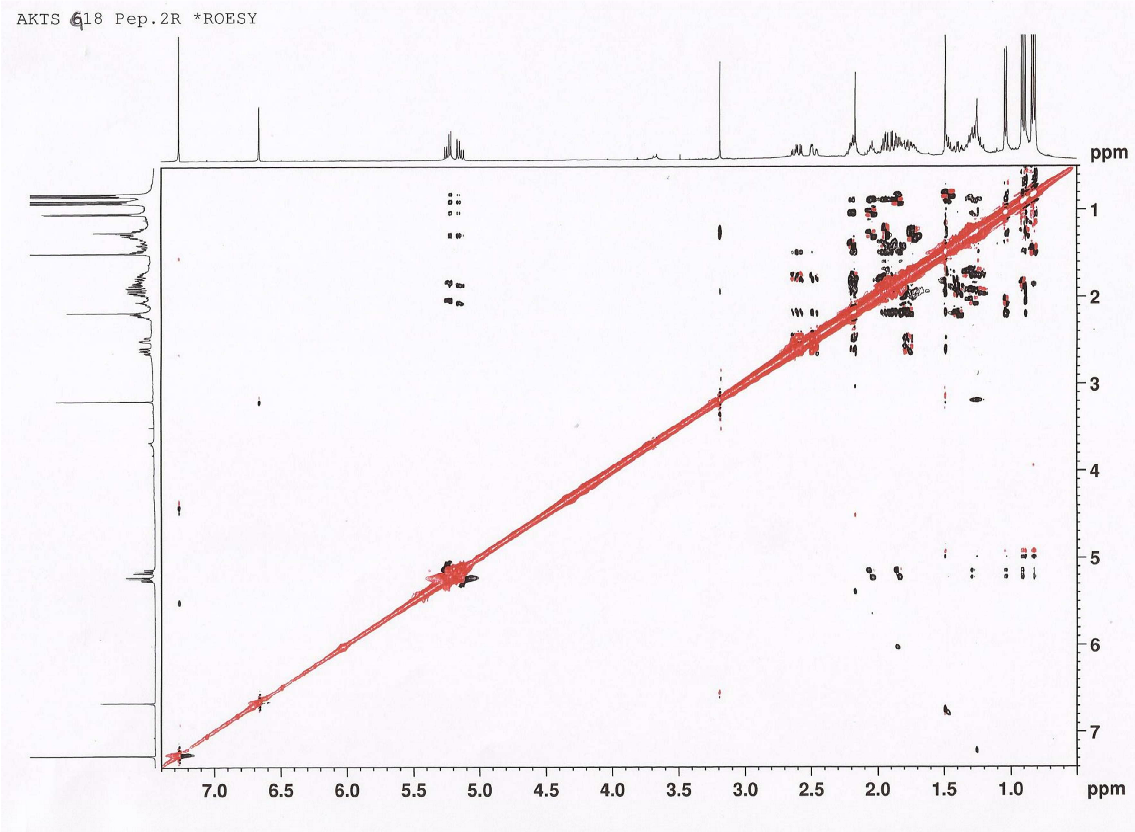

Figure S8. ^{1}H NMR spectrum of compound **2** ($CDCl_3$, 300.13 MHz).

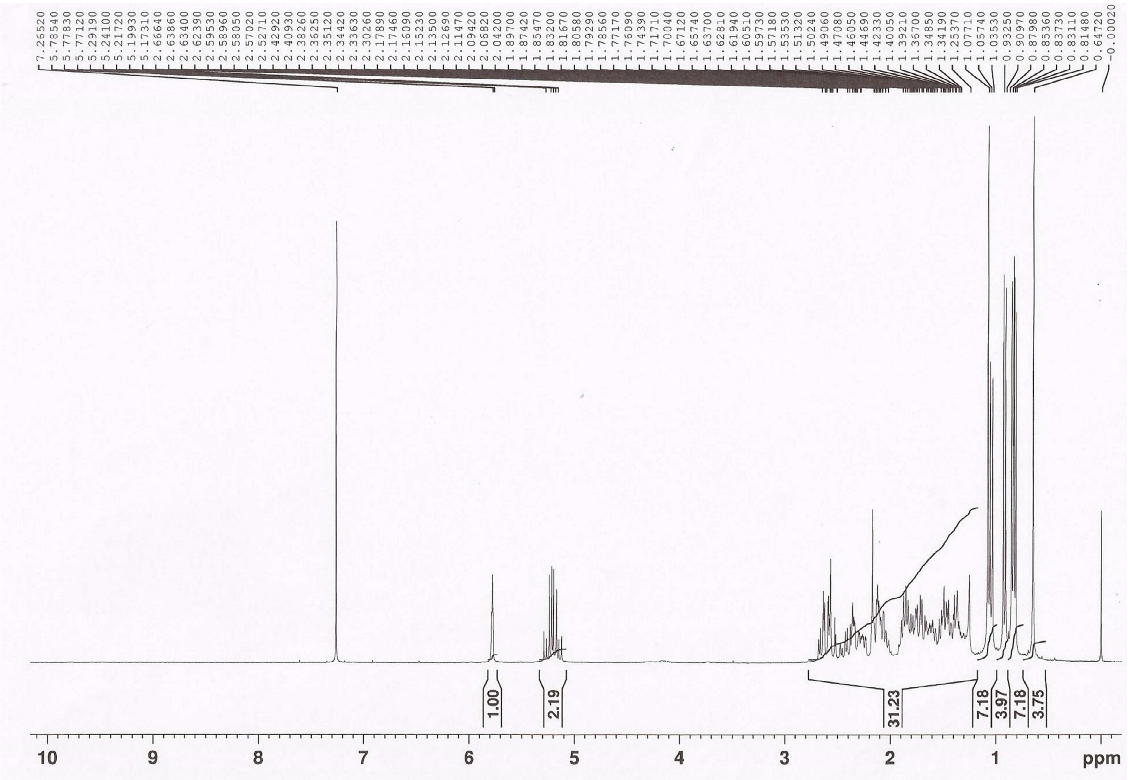

Figure S9. ^{13}C NMR spectrum of compound **2** ($CDCl_3$, 75.4 MHz).

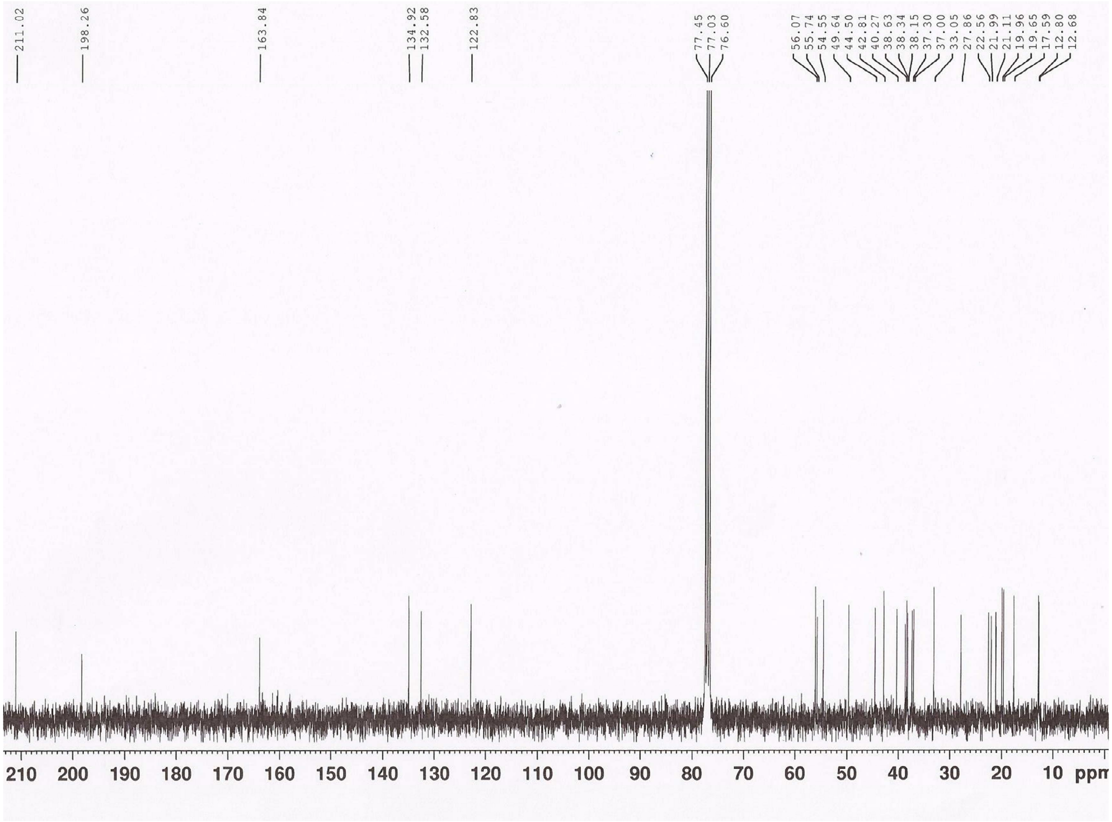

Figure S10. COSY spectrum of compound **2** ($CDCl_3$, 300.13 MHz).

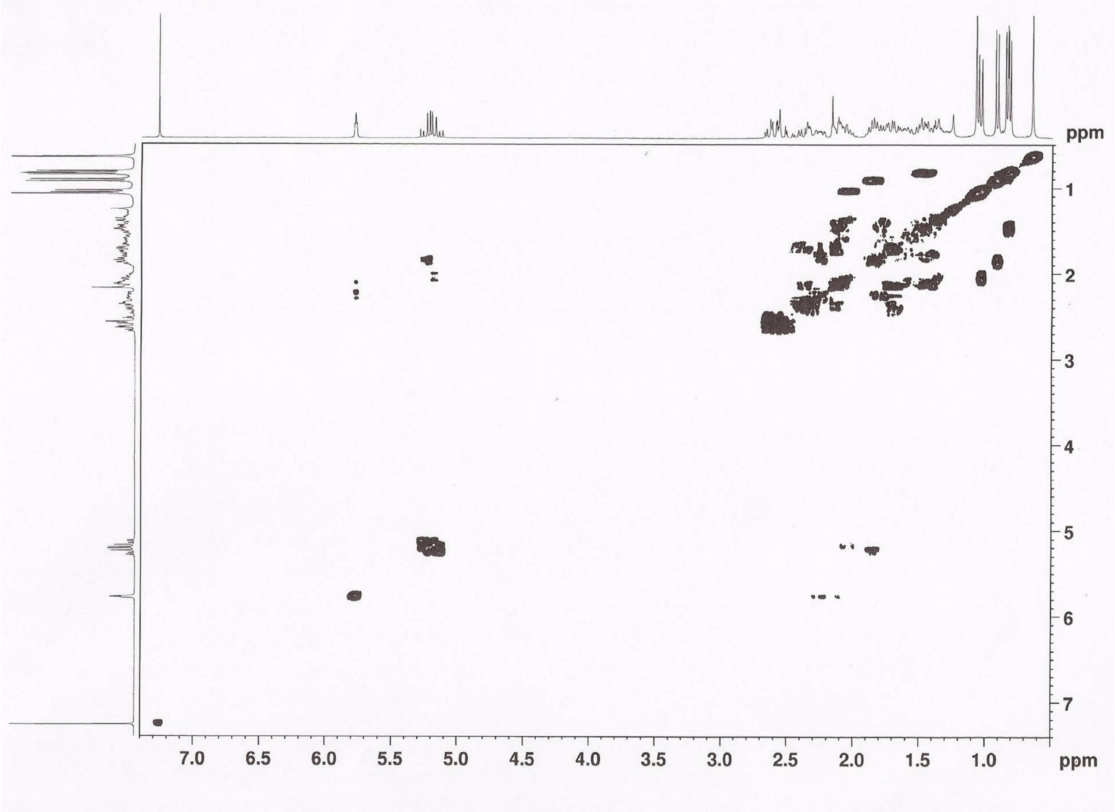

Figure S11. HSQC spectrum of compound **2** ($CDCl_3$, 300.13 MHz).

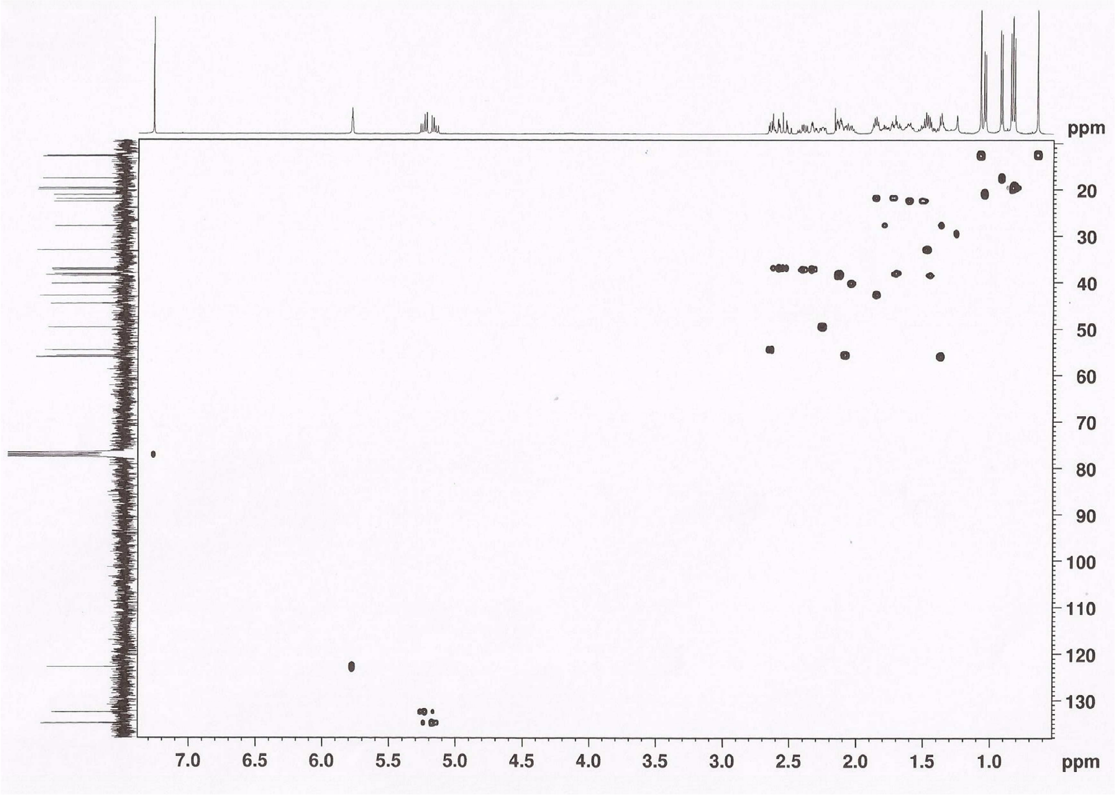

Figure S12. HMBC spectrum of compound **2** ($CDCl_3$, 300.13 MHz).

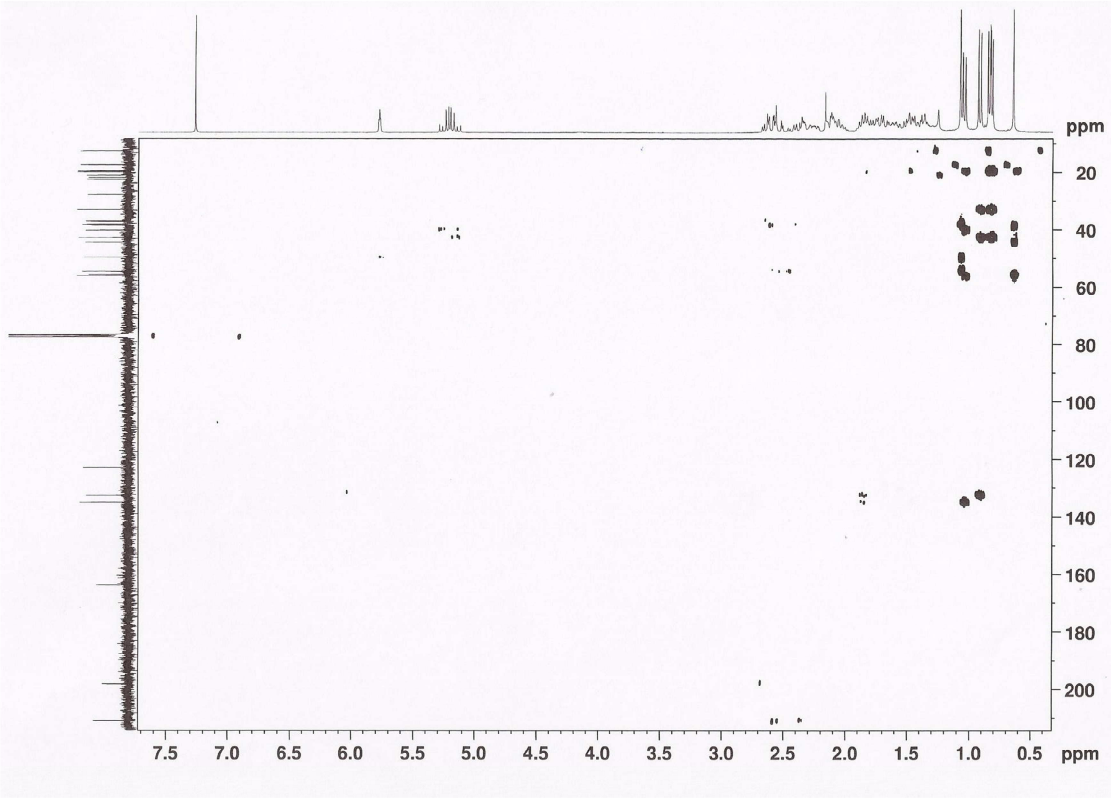

Figure S13. ^{1}H NMR spectrum of compound **3** (DMSO, 500.13 MHz).

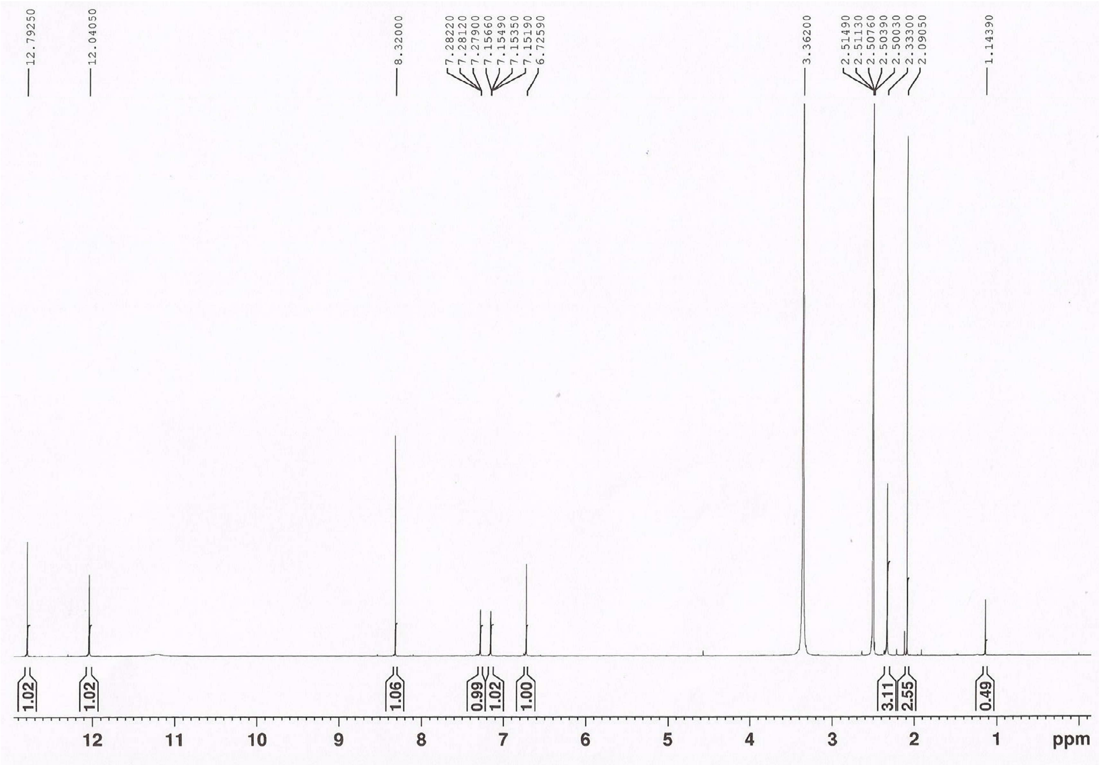

Figure S14. ^{13}C NMR spectrum of compound **3** (DMSO, 125.4 MHz).

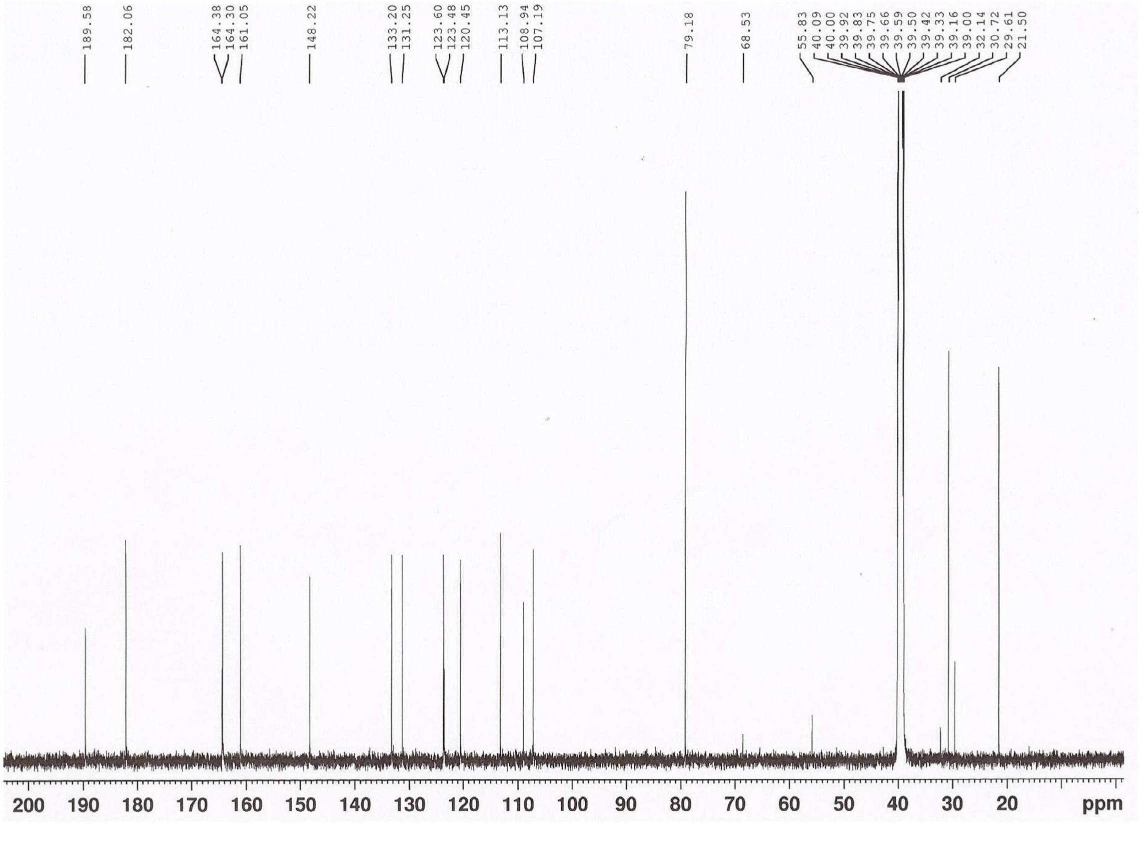

Figure S15. COSY spectrum of compound **3** (DMSO, 500.13 MHz).

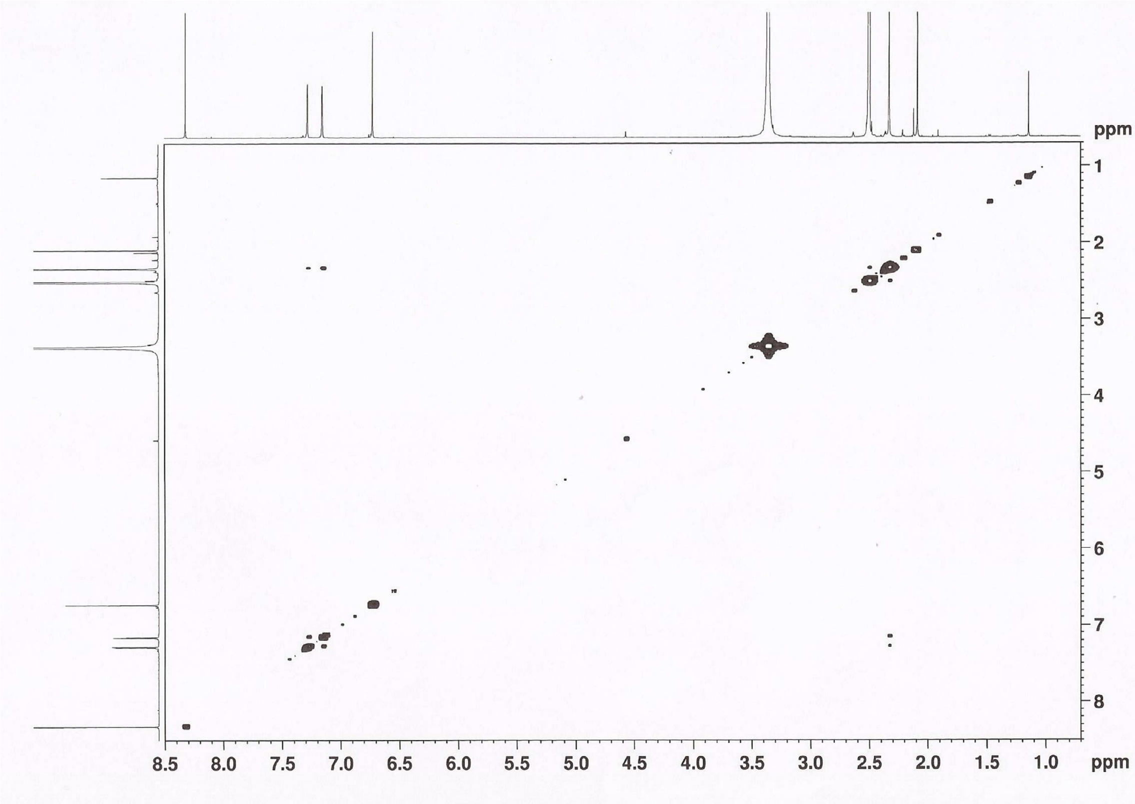

Figure S16. HSQC spectrum of compound **3** (DMSO, 500.13 MHz).

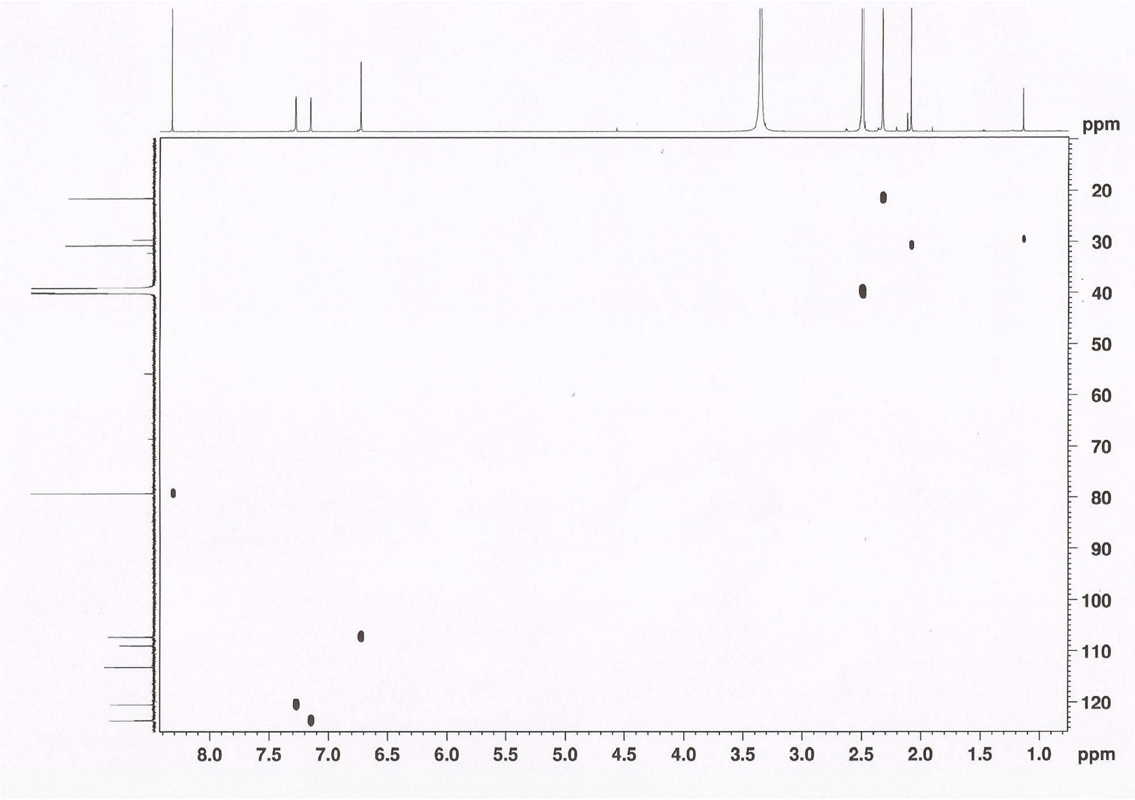

Figure S17. HMBC spectrum of compound **3** (DMSO, 500.13 MHz).

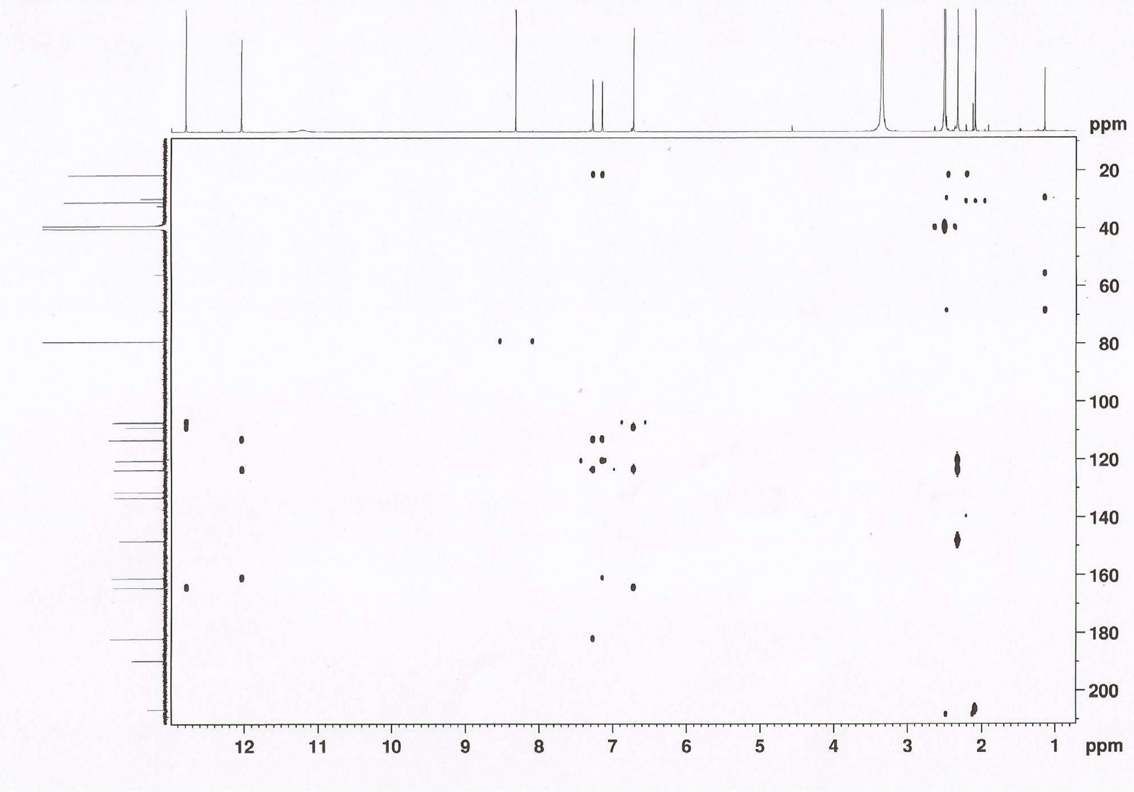

~

Table 1S

^{1}H and ^{13}C NMR ($CDCl_3$, 300.13 MHz and 75.4 MHz) and HMBC assignment for **2.**

Position	δ_C, type	δ_H, (*J* in Hz)	COSY	HMBC
1	38.2, CH_2	1.70, m		
		2.14, m		
2	37.3, CH_2	2.33, m		CO-3
		2.38, m		
3	211.0, CO	-		
4	37.0, CH_2	2.59, m	H-5	CO-3
5	54.6, CH	2.64, dd (12.8, 4.5)	H-4	
6	198.3, CO	-		
7	122.8, CH	5.78, t (2.3)	H-9, 14	C-9
8	163.8, C	-		
9	49.6, CH	2.26, m	H-7, 11	
10	38.3, C	-		
11	22.0, CH_2	1.72, m		
		1.85, m		
12	38.6, CH_2	1.45, m		
		2.14, m		
13	44.5, C	-		
14	55.7, C	2.09, m	H-9, 15	
15	22.6, CH_2	1.50, m		
		1.60, m		
16	27.9, CH_2	1.37, m		
		1.79, m		
17	56.1, CH	1.36, m	H-16, 20	
18	12.7, CH_3	0.65, s	-	
19	12.8, CH_3	1.08, s	-	C-5, 9, 10
20	40.3, CH	2.04, m	H-21, 22	
21	21.1, CH_3	1.05, d (6.6)	H-20	C-17, 20, 22
22	134.9, CH	5.16, dd (15.2, 7.9)	H-20, 23	C-20, 25
23	132.6, CH	5.25, dd (15.2, 7.2)	H-22, 24	C-20, 24
24	42.8, CH	1.85, m	H-22, 25, 26	C-22, 23
25	33.1, CH	1.48, m	H-24, 27, 28	C-23
26	17.6, CH_3	0.92, d (6.8)	H-24	C-23, 24, 25
27	20.0, CH_3	0.82, d (6.8)	H-25	C-24, 25, 26, 28
28	19.7, CH_3	0.84, d (6.8)	H-25	C-24, 25, 26, 27
